# Investigating biomechanical differences in lumbosacral transitional vertebrae among different Castellvi classifications

**DOI:** 10.3389/fbioe.2025.1700758

**Published:** 2025-11-19

**Authors:** Rui Weng, Yaoshuai Yu, Ruxia Ren, Yibin Chen, Cairui Chen, Siyuan Xie, Yikai Li, Shaoqun Zhang

**Affiliations:** 1 School of Traditional Chinese Medicine, Southern Medical University, Guangzhou, Guangdong, China; 2 The Seventh Affiliated Hospital, Sun Yat-sen University, Shenzhen, Guangdong, China; 3 Department of Classical Orthopedics and Bone Rehabilitation, Jieyang Hospital of Traditional Chinese Medicine, Jieyang, Guangdong, China; 4 School of Basic Medical Sciences, Southern Medical University, Guangzhou, Guangdong, China; 5 Shenzhen Traditional Chinese Medicine Hospital, The Fourth Clinical Medical College of Guangzhou University of Chinese Medicine, Shenzhen, Guangdong, China

**Keywords:** lumbosacral transitional vertebrae (LSTV), Castellvi classification, biomechanics, low back pain, sacroiliac joint dysfunction, sacroiliitis

## Abstract

**Objective:**

To investigate the biomechanical differences among different Castellvi classifications of lumbosacral transitional vertebrae (LSTV) based on finite element analysis.

**Methods:**

Using CT data of a healthy Asian adult male, a finite element model of the normal lumbar-pelvic complex and seven LSTV models (Castellvi types IA, IB, IIA, IIB, IIIA, IIIB, IV) were established. With bilateral acetabula fixed, 400 N axial compression (simulating body weight) and 8.0 Nm torque (simulating flexion, extension, lateral bending, rotation) were applied to each model. Differences in global displacement, maximum Mises stress of intervertebral discs and sacroiliac joints among the models were compared.

**Results:**

In terms of overall displacement, Types IIIA, IIIB, and IV were significantly lower than the normal model under all loading conditions; Types IA, IB, IIA, and IIB showed a significant reduction only under partial conditions (e.g., lateral bending, rotation). For the maximum Mises stress of intervertebral discs, Types IIIB and IV exhibited a significant reduction under all conditions; Type IIIA showed a significant reduction under all conditions except pure compression; Types IB and IIB had a significant reduction only under compression, extension, and lateral bending; Types IA and IIA showed increased stress under partial conditions (e.g., flexion, rotation). Regarding the maximum Mises stress of sacroiliac joints: the bilateral sacroiliac joints of Types IIIB and IV showed increased stress under all conditions except extension; the left sacroiliac joint of Type IIIA mainly showed an increase under most conditions, while the right side mainly showed a decrease; the bilateral sacroiliac joints of Types IB and IIB exhibited stress reduction under all conditions.

**Conclusion:**

Different Castellvi classifications of LSTV exert significant biomechanical effects on the lumbar-pelvis complex. Among them, the IIIB and IV types (including the fused left side of the IIIA type) significantly increase sacroiliac joint stress, which may contribute to sacroiliac joint dysfunction or sacroiliac joint subluxation or sacroiliitis. Types IA and IIA may easily lead to discogenic low back pain due to increased local intervertebral disc stress and uneven stress distribution. Types ⅠB and ⅡB induce minimal interference in global displacement, intervertebral disc stress, and sacroiliac joint stress, resulting in a relatively lower risk of low back pain. These results provide a biomechanical reference for the classification-based diagnosis and intervention of LSTV-related low back pain.

## 1 Introduction

Lumbosacral Transitional Vertebrae (LSTV) is a type of congenital spinal variant (Iplikcioglu and Karabag, 2024; Albano et al., 2024), characterized by anatomical variations at the lumbosacral junction, specifically sacralization of the lowest lumbar vertebra and lumbarization of the uppermost sacral vertebra. The prevalence of LSTV varies considerably in the general population. According to literature in the field of spine research, its reported incidence ranges from 4% to 35.6% (Matson et al., 2020), mainly attributed to differences in study cohorts and diagnostic methods (Matson et al., 2020; Hanhivaara et al., 2024). Notably, several recent studies have reported population-specific incidences: 16.3% in Asians (Nagata et al., 2025), 8.5% in North Americans (Verhaegen et al., 2023), 24.9% in Southern Europeans (Vinha et al., 2022), and 8.1% in Eastern Europeans (Byvaltsev et al., 2023).

Clinically, although most patients with LSTV remain asymptomatic throughout their lives, a significant subset develop symptoms such as chronic low back pain, lumbosacral radiculopathy, or accelerated degeneration of adjacent intervertebral discs and facet joints. These symptoms arise from anatomical variations, such as instability of the pseudarthrosis formed between LSTV and the sacrum, and asymmetric mechanical transmission in LSTVs with unilateral fusion or pseudarthrosis. Collectively, such conditions are termed “Bertolotti’s syndrome” (Crane et al., 2021). Currently, the mechanism underlying the association between LSTV and these symptoms remains unclear. Previous studies have suggested that the occurrence and development of LSTV may be related to intrinsic genetic factors and acquired changes in spine-pelvic biomechanics (Matson et al., 2020). Hox genes (Hox-10, Hox-11) play a vital role in vertebral segmentation and development (Carapuço et al., 2005). Vertebral development and intervertebral disc formation commence at the 4th week of embryonic development, and the fusion of vertebral bodies into the adult lumbosacral structure persists until approximately 40 years of age (Miller and Routt, 2012). The number of lumbar and sacral segments is influenced by lumbosacral load transmission during development and associated with the evolutionary process of bipedal locomotion in humans (Mahato, 2010). Studies have also indicated that LSTV results in restricted motion at the L5/S1 segment, whereas hypermobility occurs in the superior lumbar regions (Golubovsky et al., 2020). Structural abnormalities at the lumbosacral junction can disrupt the normal physiological load transmission of the spine, leading to increased stress concentration in adjacent spinal structures and subsequent degenerative changes (Zhu et al., 2025; Abul, 2024). For a long time, lumbosacral biomechanical disturbances caused by LSTV have been considered potentially associated with chronic low back pain and spinal degenerative lesions (Bhagchandani et al., 2024; Hanhivaara et al., 2022; Hanhivaara et al., 2020; Desai et al., 2024), thus making it a critical focus in spinal research and clinical practice.

Currently, LSTV exhibit different types of anatomical variations. The classification system proposed by Castellvi et al. is currently widely used for LSTV typing (Castellvi et al., 1984), which categorizes LSTV into four types (Type I to Type IV) based on the morphology of transverse processes and their degree of fusion with the sacrum. Existing studies have suggested an association between LSTV and chronic low back pain as well as sacroiliac joint dysfunction (Illeez et al., 2018), though the underlying mechanism remains unclear (Vinha et al., 2022; Hanhivaara et al., 2022). We hypothesize that the potential association between LSTV, low back pain, and sacroiliac joint dysfunction may be related to the different anatomical variants of LSTV. Given that different anatomical variants exert varying effects on the stress of adjacent spinal structures, certain types of LSTV may induce pelvic instability or abnormally increased stress on lumbar intervertebral discs and sacroiliac joints. Such abnormal stress elevation is likely to increase the risk of low back pain and sacroiliac joint dysfunction. In contrast, other types of LSTV may not affect the aforementioned parameters and thus do not elevate the risk of these conditions. However, these hypotheses currently lack validation from relevant biomechanical studies. Therefore, this study aims to compare the stress differences on structures such as pelvic stability, lumbar intervertebral discs, and sacroiliac joints between finite element models of different LSTV classifications and the normal model through finite element analysis. Furthermore, it aims to provide biomechanical insights into the potential impacts of different LSTV types on the development of low back pain and sacroiliac joint dysfunction.

## 2 Materials and methods

### 2.1 Data selection

Based on the computed tomography (CT) data of a 28-year-old healthy Asian male volunteer (178 cm, 85 kg), a three-dimensional finite element model of the lumbar-pelvis was constructed. The volunteer was confirmed by CT examination to have no diseases such as lumbar spine and pelvic fractures, deformities, tumors, or ankylosing spondylitis. This study has been approved by the Medical Ethics Committee of Shenzhen Traditional Chinese Medicine Hospital (K2023-086-01), and the volunteer has provided a written informed consent form.

Continuous scanning was performed using a 64-slice spiral CT scanner (SOMATOM Definition AS+). The CT scanning parameters were as follows: tube voltage of 120 kV, tube current of 220 mA, slice thickness of 1.25 mm, and slice interval of 0.75 mm. The scanning range extended from the upper edge of the 5th lumbar vertebra to the upper end of the femur, including the ischial tuberosities and pubic bones. The data were saved in DICOM format.

### 2.2 Establishment of a three-dimensional finite element reference model of the lumbar -pelvis

**FIGURE 1:**
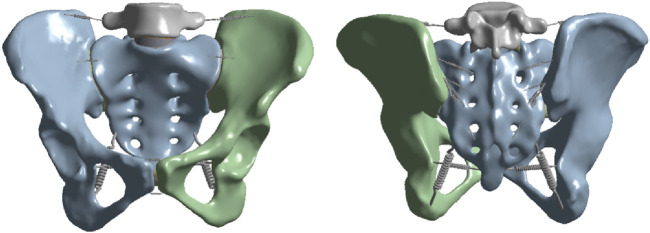
Schematic diagrams of the anterior and posterior views of the lumbar-pelvic finite element model and the surrounding ligaments.

The CT scan data were imported into the medical image processing software Mimics 19.0 (Materialize, Belgium) for preprocessing. To accurately extract the bony structures in the lumbar-pelvic region (including lumbar vertebrae, sacrum, ilium, etc.), a threshold range of 720–3,000 Hounsfield Units (HU) was set based on the HU distribution characteristics of bones in this region on CT images to extract the target bone tissues and generate a mask. Additionally, incomplete scanned regions in the CT images were supplemented and repaired. Subsequently, the data were converted into a three-dimensional model of the human lumbar-pelvis, which was exported in STL format and imported into the reverse engineering software Geomagic Studio 2013 (Geomagic, United States). In this software, the “Mesh Doctor” function was used to smooth the model surface, repair holes, and remove spikes. Then, in the precise surface stage, the probabilistic curvature method was adopted to fit the model into a precise surface (Weng et al., 2022), which was imported into SolidWorks 2017 (Dassault Systems Corporation, France) in STP format. In this software, corresponding structures were constructed, including cortical bone, cancellous bone, endplates, intervertebral discs, facet joints, pubic symphysis, sacroiliac joints, and surrounding ligaments (Zhang et al., 2022). The intervertebral disc mainly consists of two parts: the annulus fibrosus and the nucleus pulposus, with a volume ratio of approximately 6:4 (Weng et al., 2025). The surrounding ligaments mainly include the anterior sacroiliac ligament, long posterior sacroiliac ligament, short posterior sacroiliac ligament, interosseous sacroiliac ligament, sacrospinous ligament, sacrotuberous ligament, superior pubic ligament, arcuate pubic ligament, iliolumbar ligament, posterior longitudinal ligament, anterior longitudinal ligament, interspinous ligament, supraspinous ligament, ligamentum flavum, etc. (as shown in Figure 1) (Henyš et al., 2022; Hammer et al., 2013; Yang et al., 2020). These ligaments were defined as tension-only spring elements to minimize other interferences and simulate the mechanical properties of ligaments, and corresponding stiffness was assigned to them.

Subsequently, ANSYS Workbench 2023R1 (ANSYS, Inc., United States) was used to set the material properties of the model (Zhang et al., 2022; Gierig et al., 2021; Luo et al., 2025; Kumaran et al., 2023), with specific parameters shown in Table 1. In this software, contact relationships were defined to simulate the interactions between various structures of the lumbar-pelvis. Bonded contacts were set for the connections between cortical bone and cancellous bone, endplates and intervertebral discs, intervertebral discs and upper/lower vertebral bodies, as well as between ligaments and bones. Frictional contacts with a friction coefficient of 0.1 were set for the facet joints and sacroiliac joints (Gierig et al., 2021). The above contact settings were intended to closely approximate the mechanical behaviors between various structures under physiological conditions.

**TABLE 1:** Material properties of each material in the lumbar-pelvic three-dimensional finite element model.

Components	Young’s modulus (MPa)	Poisson’s ratio	Constitutive relation	Element type
Vertebral cortical bone	10,000	0.3	Isotropic, elastic	4 nodes tetrahedral element (C3D4)
Vertebral cancellous bone	100	0.2	Isotropic, elastic	4 nodes tetrahedral element (C3D4)
Pelvic cortical bone	17,000	0.3	Isotropic, elastic	4 nodes tetrahedral element (C3D4)
Pelvic cancellous bone	10	0.2	Isotropic, elastic	4 nodes tetrahedral element (C3D4)
Ground substance of annulus fibrosis	C_10_ = 0.035 K_1_ = 0.296 K_2_ = 65	-	Anisotropic, hyperelastic	8 nodes brick element (C3D8)
Nucleus pulposus	4	0.49	Isotropic, elastic	4 nodes tetrahedral element (C3D4)
Endplate	100	0.4	Isotropic, elastic	4 nodes tetrahedral element (C3D4)
Ligaments	Nonlinear stress ‐ strain curves	-	Hypoelastic	Tension ‐ only, truss elements (T3D2)
Apophyseal joints	-	-	Nonlinear soft contact	4 nodes tetrahedral element (C3D4)
Sacroiliac joints	-	-	Nonlinear soft contact	4 nodes tetrahedral element (C3D4)
Pubic symphysis	Pressure ‐ overclosure	-	Nonlinear soft contact	4 nodes tetrahedral element (C3D4)

### 2.3 Validation of the three-dimensional finite element reference model

For the validation of this finite element reference model, both acetabular fossae were fully fixed, and an axial compressive force of 400 N was applied downward to the upper end of L5 to simulate body weight. In addition, a torque of 7.5 Nm was applied to the upper surface of the L5 vertebra to induce flexion, extension, lateral bending, and axial rotation, respectively (Lindsey et al., 2014). The validity of the model was verified by measuring the range of motion (ROM) of the sacroiliac joint and comparing it with the results of other *in vitro* studies or finite element studies.

Additionally, to verify mesh convergence, four mesh models with different densities were designed in this study. The element size of the models was gradually reduced by a geometric ratio of 0.7, starting from an initial size of 5.8 mm, followed by sequential adjustments to 4.1 mm, 2.9 mm, and 2.0 mm. Meanwhile, local mesh refinement was implemented in the region where the intervertebral disc is located. The maximum compressive stress of the intervertebral disc was selected as the index for judging convergence. Under the same load condition (a downward axial compressive force of 400 N applied) and boundary constraints, the stress values of the four mesh schemes were calculated respectively. Subsequently, the relative error rate between two adjacent mesh schemes was computed; convergence was determined when this error rate dropped below 5% (Lin et al., 2023), which also indicates that the mesh density at this point meets the requirements of calculation accuracy.

### 2.4 Construction of LSTV models with different Castellvi classifications

**FIGURE 2:**
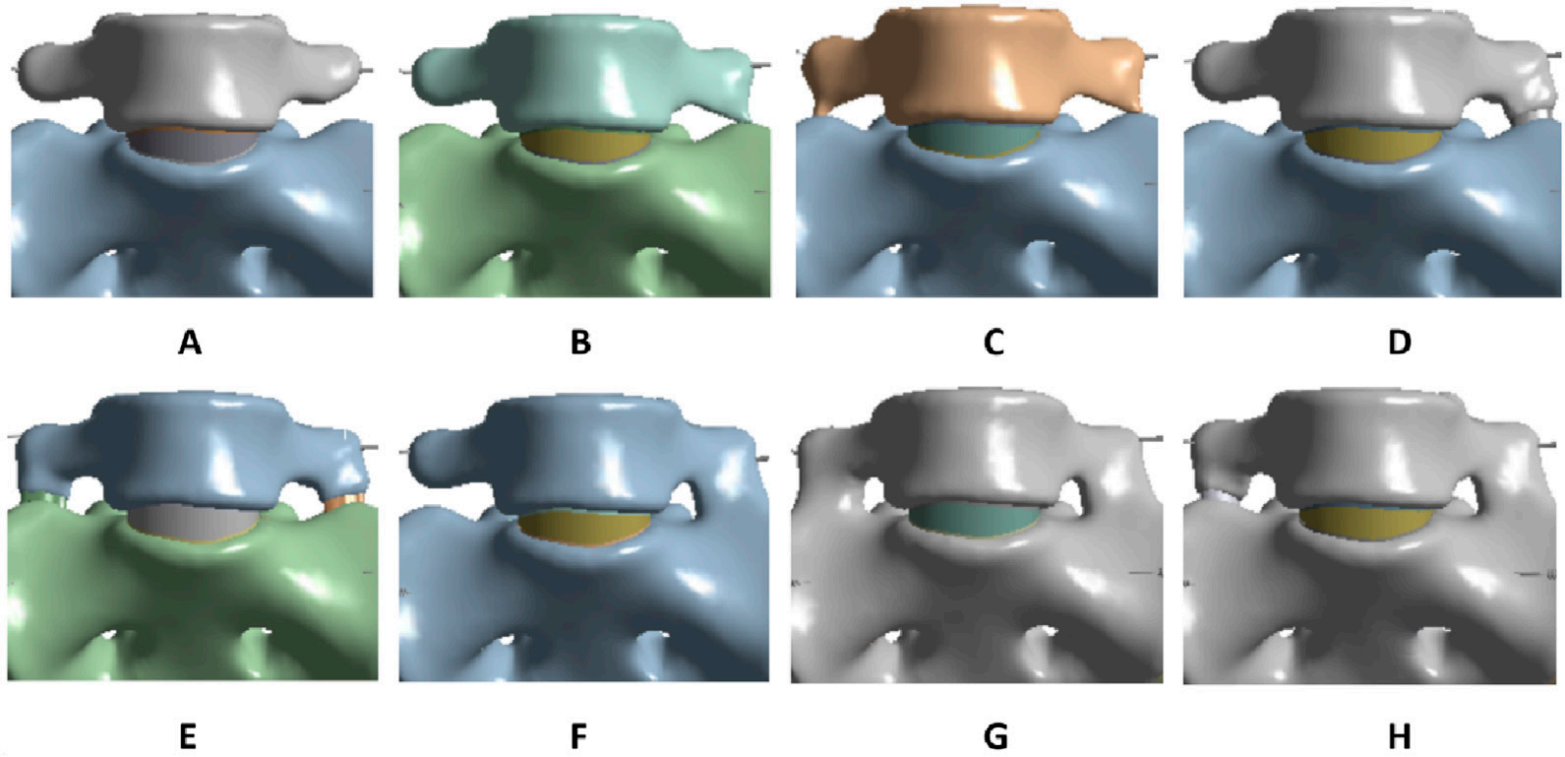
Anterior views of lumbar transitional vertebrae with different types **(A–H)**.

The validated reference model was used as the normal model (Figure 2A). Based on the LSTV classification proposed by Castellvi et al. (1984), seven different types of LSTV models were constructed on the basis of the normal model using SolidWorks 2017 software. The first type (Type ⅠA) is characterized by unilateral lumbar transverse process hypertrophy, with a craniocaudal diameter of at least 19 mm (Figure 2B). The second type (Type ⅠB) presents with bilateral lumbar transverse process hypertrophy, where both craniocaudal diameters exceed 19 mm (Figure 2C). The third type (Type ⅡA) refers to incomplete lumbar sacralization/sacral lumbarization, featuring unilateral enlarged lumbar transverse processes that form synovial joints with the sacrum (Figure 2D). The fourth type (Type ⅡB) is defined by bilateral enlarged lumbar transverse processes, both of which form synovial joints with the sacrum (Figure 2E). The fifth type (Type ⅢA) represents unilateral lumbar sacralization/sacral lumbarization, accompanied by complete osseous fusion between the transverse process and the sacrum (Figure 2F). The sixth type (Type ⅢB) denotes bilateral lumbar sacralization/sacral lumbarization, with complete osseous fusion of both transverse processes to the sacrum (Figure 2G). The seventh type (Type Ⅳ) is a mixed type: one side shows incomplete lumbar sacralization/sacral lumbarization (manifested as an enlarged transverse process forming a synovial joint with the sacrum), while the other side exhibits complete lumbar sacralization/sacral lumbarization (characterized by complete osseous fusion between the transverse process and the sacrum) (Figure 2H).

**FIGURE 3:**
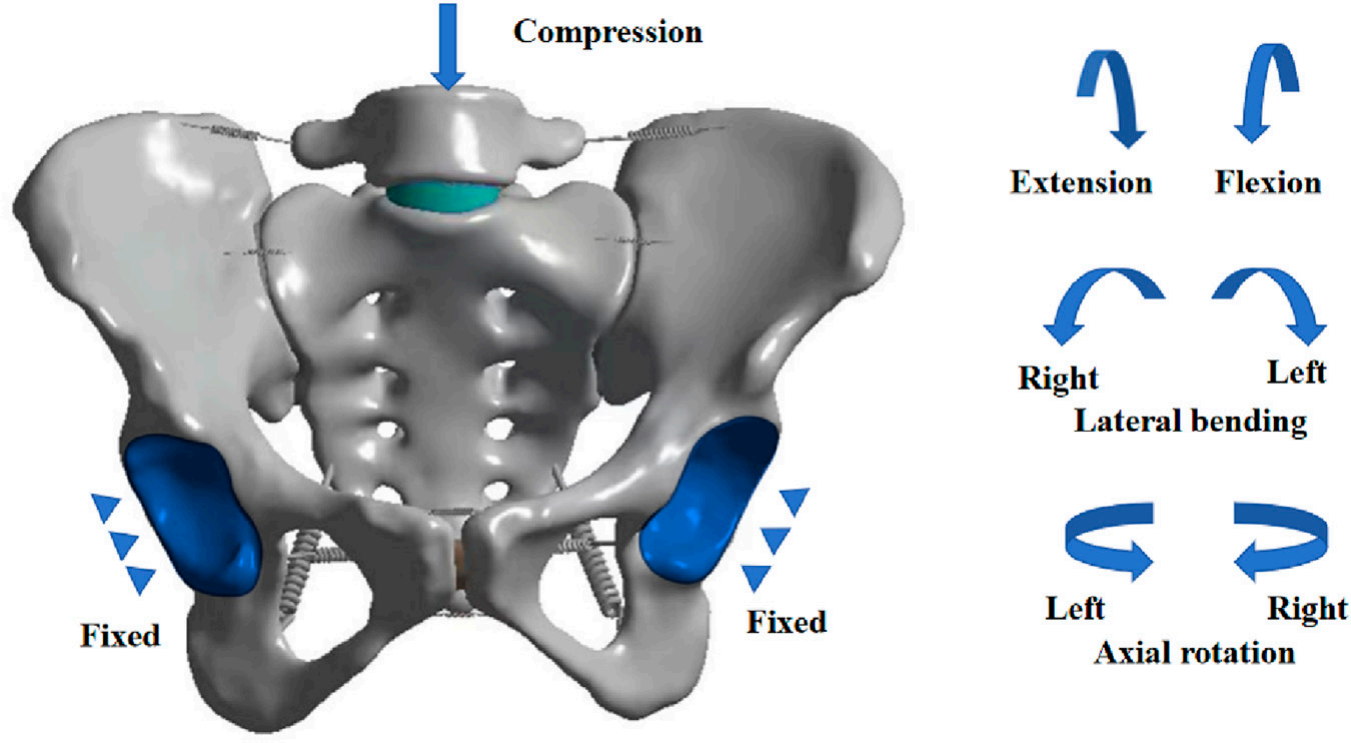
Schematic diagram of boundary loading conditions for the finite element model.

In SolidWorks 2017, based on the characteristics of the LSTV classification by Castellvi et al., we modified the corresponding structures of the L5 vertebra and sacrum by constructing solids and editing features—including adjusting dimensions, modifying contour sketches, and deleting features—to match the morphologies of different LSTV types. Except for the aforementioned specific structural differences, all other anatomical structures of the newly constructed models of various LSTV, such as intervertebral discs, sacroiliac joints, and ligaments, were consistent with those of the normal model. To compare the biomechanical properties among different types, a unified mechanical loading protocol was applied to all models in the ANSYS Workbench software (Figure 3):

1. The geometric surfaces of the bilateral acetabular fossae were selected, and a Fixed Support was applied to them to restrict all degrees of freedom
2. Taking the upper surface of the L5 vertebra as the acting surface, a distributed axial compressive force of 400 N was applied to it to simulate body weight.
3. Meanwhile, the central point of the upper surface of the L5 vertebra was taken as the reference node, and this node was coupled with the surrounding area. A torque of 8.0 Nm was applied to this node around specific axes: flexion/extension around the coronal axis, lateral bending around the sagittal axis, and axial rotation around the longitudinal axis.

Through computational analysis, the maximum Mises stress in the intervertebral discs and sacroiliac joints, as well as the overall displacement, were compared among the models of different types.

## 3 Results

### 3.1 Validation results of the finite element model

**FIGURE 4:**
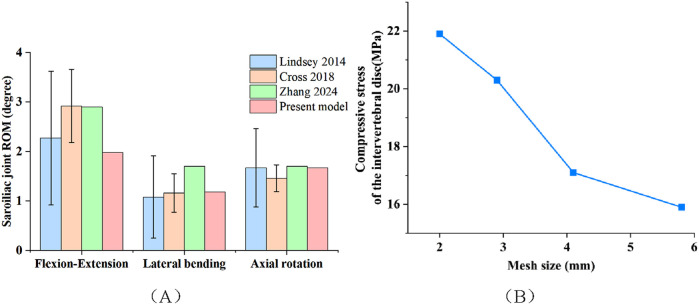
**(A)** The ROM for flexion–extension, lateral bending, and axial rotation in the three-dimensional finite element model. **(B)** Mesh convergence test for the normal model.

The ROM results of the finite element reference model are shown in Figure 4A. When compared with the results of *in vitro* cadaver experiments and previous finite element studies (Lindsey et al., 2014; Cross et al., 2018; Zhang et al., 2024), the ROM of this model showed good consistency with the reference data. This indicates that the model validation was effective, and the model can be used for experimental analysis. A mesh convergence study was conducted using four mesh sizes (5.8 mm, 4.1 mm, 2.9 mm, and 2.0 mm). When the mesh sizes were 2.9 mm and 2.0 mm, the difference in the maximum compressive stress of the intervertebral disc was within 5% (Figure 4B). Considering both calculation accuracy and efficiency, a mesh size of 2.9 mm was selected for this study, which included a total of 473,164 elements and 248,525 nodes.

### 3.2 Overall displacement of different models

**FIGURE 5:**
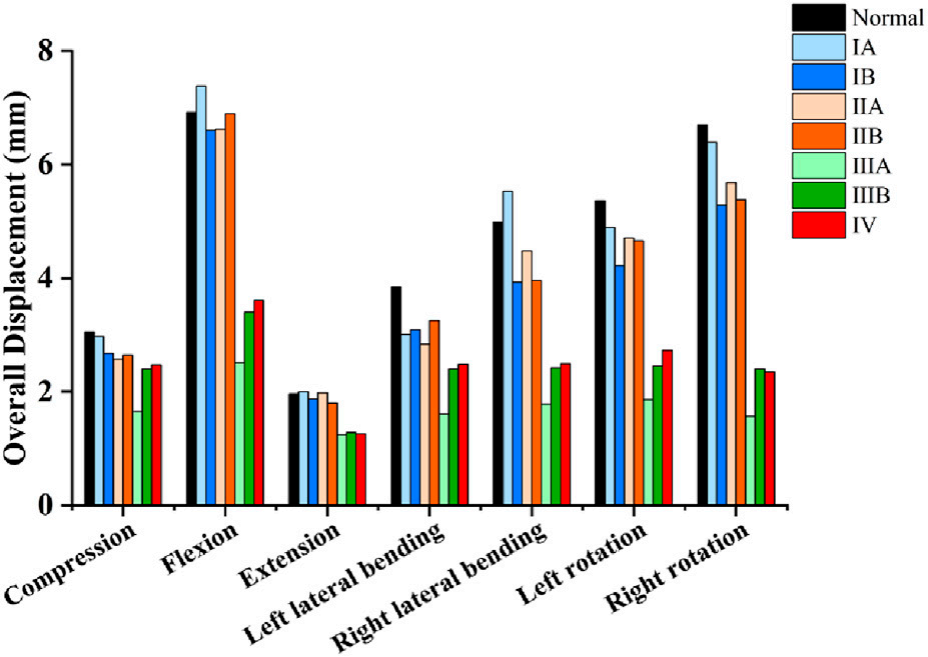
Comparison of the overall displacement of different models under various loading conditions.

The overall displacement of each model under various loading conditions is presented in Figure 5 and Table 2. For models ⅠA, ⅠB, ⅡA, and ⅡB, no significant differences in overall displacement were observed compared to the Normal model under flexion and extension. Specifically, model ⅠA showed no significant displacement differences from the Normal model under pure compression, left rotation, or right rotation; however, its displacement decreased under left lateral bending and increased under right lateral bending compared to the Normal model. Additionally, the overall displacement of models ⅠB, ⅡA, and ⅡB all decreased compared to the Normal model under pure compression, left/right lateral bending, and left/right rotation. In contrast, models ⅢA, ⅢB, and Ⅳ showed a greater magnitude of decrease in overall displacement compared to the Normal model under pure compression, flexion, extension, left/right lateral bending, and left/right rotation.

**TABLE 2:** Overall displacement values (mm) and percentage changes versus the normal model under various loading conditions.

Loading condition	Normal	ⅠA (% change)	ⅠB (% change)	ⅡA (% change)	ⅡB (% change)	ⅢA (% change)	ⅢB (% change)	Ⅳ (% change)
Compression	3.05	2.97 (N)	2.67 (−12.46%)	2.57 (−15.74%)	2.64 (−13.44%)	1.65 (−45.90%)	2.40 (−21.31%)	2.47 (−19.02%)
Flexion	6.92	7.38 (N)	6.60 (N)	6.62 (N)	6.89 (N)	2.51 (−63.73%)	3.40 (−50.87%)	3.61 (−47.83%)
Extension	1.96	2.00 (N)	1.87 (N)	1.98 (N)	1.80 (N)	1.24 (−36.73%)	1.28 (−34.69%)	1.26 (−35.71%)
Left lateral bending	3.85	3.01 (−21.82%)	3.09 (−19.74%)	2.84 (−26.23%)	3.25 (−15.58%)	1.61 (−58.18%)	2.40 (−37.66%)	2.48 (−35.58%)
Right lateral bending	4.98	5.53 (+11.04%)	3.93 (−21.08%)	4.48 (−10.04%)	3.96 (−20.48%)	1.78 (−64.26%)	2.42 (−51.41%)	2.49 (−49.99%)
Left rotation	5.36	4.89 (N)	4.22 (−21.27%)	4.71 (−12.13%)	4.66 (−13.06%)	1.86 (−65.30%)	2.45 (−54.29%)	2.73 (−49.07%)
Right rotation	6.69	6.39 (N)	5.28 (−21.08%)	5.68 (−15.10%)	5.38 (−19.58%)	1.57 (−76.53%)	2.40 (−64.13%)	2.35 (−64.87%)

“% Change” refers to the percentage change versus the Normal model, calculated as (Model Value − Normal Model Value)/Normal Model Value × 100%.

“−” = Reduction; “+” = Increase.

“N” indicates a percentage change with absolute value <10%.

### 3.3 Comparison of maximum mises stresses among different models

**FIGURE 6:**
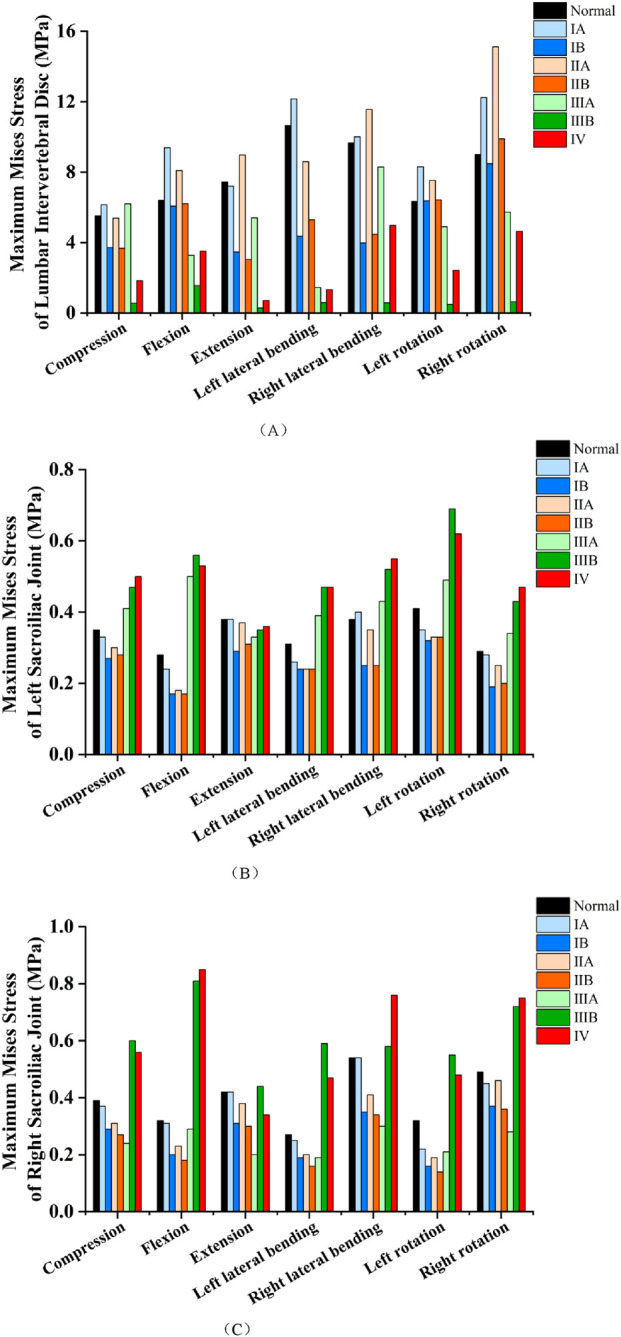
**(A)** Comparison of the maximum Mises stresses in the intervertebral discs of different models under various loading conditions. **(B)** Comparison of the maximum Mises stresses in the left sacroiliac joint of different models under various loading conditions. **(C)** Comparison of the maximum Mises stresses in the right sacroiliac joint of different models under various loading conditions.

The maximum Mises stress in the intervertebral discs across all models under various loading conditions is presented in Figure 6A and Table 3. Model ⅠA displayed no significant differences in intervertebral disc maximum Mises stress compared with the Normal model under extension and right lateral bending. However, increases were noted under pure compression, flexion, left lateral bending, and left/right rotation. For model ⅡA, no significant difference in intervertebral disc maximum Mises stress was detected compared with the Normal model under pure compression, while a reduction was observed under left lateral bending. In contrast, increases were noted under flexion, extension, right lateral bending, and left/right rotation. Models ⅠB and ⅡB showed no significant differences in intervertebral disc maximum Mises stress from the Normal model under flexion and left/right rotation. Conversely, their stress was reduced under pure compression, extension, and left/right lateral bending. In model ⅢA, the maximum Mises stress in the intervertebral discs was slightly increased relative to the Normal model under pure compression, whereas decreases were observed under flexion, extension, left/right lateral bending, and left/right rotation. Models ⅢB and Ⅳ exhibited significantly reduced maximum Mises stress in the intervertebral discs under all seven loading conditions: pure compression, flexion, extension, left/right lateral bending, and left/right rotation. Among these, model ⅢB showed the most pronounced reductions.

**TABLE 3:** Maximum mises stress values of intervertebral discs (MPa) and percentage changes versus the normal model under various loading conditions.

Loading condition	Normal	ⅠA (% change)	ⅠB (% change)	ⅡA (% change)	ⅡB (% change)	ⅢA (% change)	ⅢB (% change)	Ⅳ (% change)
Compression	5.52	6.15 (+11.41%)	3.71 (−32.79%)	5.39 (N)	3.68 (−33.33%)	6.20 (+12.32%)	0.56 (−89.85%)	1.84 (−66.67%)
Flexion	6.40	9.38 (+46.56%)	6.07 (N)	8.09 (+26.41%)	6.21 (N)	3.27 (−48.91%)	1.55 (−75.78%)	3.51 (−45.16%)
Extension	7.45	7.21 (N)	3.46 (−53.56%)	8.97 (+20.40%)	3.04 (−59.19%)	5.41 (−27.38%)	0.29 (−96.11%)	0.71 (−90.47%)
Left lateral bending	10.65	12.16 (+14.18%)	4.36 (−59.06%)	8.59 (−19.34%)	5.30 (−50.23%)	1.45 (−86.39%)	0.60 (−94.37%)	1.33 (−87.42%)
Right lateral bending	9.67	10.01 (N)	3.98 (−58.84%)	11.56 (+19.54%)	4.47 (−53.77%)	8.29 (−14.27%)	0.58 (−93.90%)	4.97 (−48.60%)
Left rotation	6.34	8.30 (+30.91%)	6.37 (N)	7.53 (+18.77%)	6.42 (N)	4.90 (−22.71%)	0.49 (−92.27%)	2.43 (−61.67%)
Right rotation	9.00	12.24 (+36.00%)	8.48 (N)	15.12 (+68.00%)	9.89 (N)	5.73 (−36.33%)	0.64 (−92.89%)	4.64 (−48.44%)

The maximum Mises stress in the left sacroiliac joint across all models under various loading conditions is presented in Figure 6B and Table 4. In model ⅠA, the maximum Mises stress showed no significant difference from the Normal model under pure compression, extension, right lateral bending, or right rotation. Conversely, a slight decrease was noted under flexion, left lateral bending, and left rotation. Model ⅡA displayed no significant difference in the maximum Mises stress of the left sacroiliac joint compared with the Normal model under extension and right lateral bending, but a reduction was observed under pure compression, flexion, left lateral bending, and left/right rotation. Models ⅠB and ⅡB consistently demonstrated reduced maximum Mises stress in the left sacroiliac joint relative to the Normal model across all evaluated loading conditions, including pure compression, flexion, extension, left/right lateral bending, and left/right rotation. Model ⅢA exhibited a reduction in the maximum Mises stress of the left sacroiliac joint relative to the Normal model under extension, whereas an increase was observed under pure compression, flexion, left/right lateral bending, and left/right rotation. For models ⅢB and Ⅳ, no significant differences in the maximum Mises stress of the left sacroiliac joint were detected compared with the Normal model under extension. However, their stress was significantly increased under pure compression, flexion, left/right lateral bending, and left/right rotation.

**TABLE 4:** Maximum mises stress values of left sacroiliac joint (MPa) and percentage changes versus the normal model under various loading conditions.

Loading condition	Normal	ⅠA (% change)	ⅠB (% change)	ⅡA (% change)	ⅡB (% change)	ⅢA (% change)	ⅢB (% change)	Ⅳ (% change)
Compression	0.35	0.33 (N)	0.27 (−22.86%)	0.30 (−14.29%)	0.28 (−20.00%)	0.41 (+17.14%)	0.47 (+34.29%)	0.50 (+42.86%)
Flexion	0.28	0.24 (−14.29%)	0.17 (−39.29%)	0.18 (−35.71%)	0.17 (−39.29%)	0.50 (+78.57%)	0.56 (+100.00%)	0.53 (+89.29%)
Extension	0.38	0.38 (N)	0.29 (−23.68%)	0.37 (N)	0.31 (−18.42%)	0.33 (−13.16%)	0.35 (N)	0.36 (N)
Left lateral bending	0.31	0.26 (−16.13%)	0.24 (−22.58%)	0.24 (−22.58%)	0.24 (−22.58%)	0.39 (+25.81%)	0.47 (+51.61%)	0.47 (+51.61%)
Right lateral bending	0.38	0.40 (N)	0.25 (−34.21%)	0.35 (N)	0.25 (−34.21%)	0.43 (+13.16%)	0.52 (+36.84%)	0.55 (+44.74%)
Left rotation	0.41	0.35 (−14.63%)	0.32 (−21.95%)	0.33 (−19.51%)	0.33 (−19.51%)	0.49 (+19.51%)	0.69 (+68.29%)	0.62 (+51.22%)
Right rotation	0.29	0.28 (N)	0.19 (−34.48%)	0.25 (−13.79%)	0.20 (−31.03%)	0.34 (+17.24%)	0.43 (+48.28%)	0.47 (+62.07%)

The maximum Mises stress in the right sacroiliac joint across all models under various loading conditions is presented in Figure 6C and Table 5. For model ⅠA, no significant differences in the maximum Mises stress of the right sacroiliac joint were observed compared with the Normal model under pure compression, flexion, extension, left/right lateral bending, or right rotation. However, a reduction in stress was noted under left rotation. In model ⅡA, the maximum Mises stress did not differ significantly from that of the Normal model under extension and right rotation, whereas decreases were observed under pure compression, flexion, left/right lateral bending, and left rotation. Models ⅠB and ⅡB consistently exhibited reduced maximum Mises stress in the right sacroiliac joint relative to the Normal model across all evaluated loading conditions: pure compression, flexion, extension, left/right lateral bending, and left/right rotation. For model ⅢA, no significant difference in the maximum Mises stress of the right sacroiliac joint was detected compared with the Normal model under flexion. Conversely, stress reductions were observed under pure compression, extension, left/right lateral bending, and left/right rotation. In model ⅢB, the maximum Mises stress of the right sacroiliac joint showed no significant difference from the Normal model under extension and right lateral bending; however, increases were noted under pure compression, flexion, left lateral bending, and left/right rotation. Model Ⅳ displayed a reduction in the maximum Mises stress of the right sacroiliac joint compared with the Normal model under extension. In contrast, stress increases were observed under pure compression, flexion, left/right lateral bending, and left/right rotation.

**TABLE 5:** Maximum mises stress values of right sacroiliac joint (MPa) and percentage changes versus the normal model under various loading conditions.

Loading condition	Normal	ⅠA (% change)	ⅠB (% change)	ⅡA (% change)	ⅡB (% change)	ⅢA (% change)	ⅢB (% change)	Ⅳ (% change)
Compression	0.39	0.37 (N)	0.29 (−25.64%)	0.31 (−20.51%)	0.27 (−30.77%)	0.24 (−38.46%)	0.60 (+53.85%)	0.56 (+43.59%)
Flexion	0.32	0.31 (N)	0.20 (−37.50%)	0.23 (−28.13%)	0.18 (−43.75%)	0.29 (N)	0.81 (+153.13%)	0.85 (+165.63%)
Extension	0.42	0.42 (N)	0.31 (−26.19%)	0.38 (N)	0.30 (−28.57%)	0.20 (−52.38%)	0.44 (N)	0.34 (−19.05%)
Left lateral bending	0.27	0.25 (N)	0.19 (−29.63%)	0.20 (−25.93%)	0.16 (−40.74%)	0.19 (−29.63%)	0.59 (+118.52%)	0.47 (+74.07%)
Right lateral bending	0.54	0.54 (N)	0.35 (−35.19%)	0.41 (−24.07%)	0.34 (−37.04%)	0.30 (−44.44%)	0.58 (N)	0.76 (+40.74%)
Left rotation	0.32	0.22 (−31.25%)	0.16 (−50.00%)	0.19 (−40.63%)	0.14 (−56.25%)	0.21 (−34.38%)	0.55 (+71.88%)	0.48 (+50.00%)
Right rotation	0.49	0.45 (N)	0.37 (−24.49%)	0.46 (N)	0.36 (−26.53%)	0.28 (−42.86%)	0.72 (+46.94%)	0.75 (+53.06%)

“% Change” refers to the percentage change versus the Normal model, calculated as (Model Value − Normal Value)/Normal Value × 100%.

“−” = Reduction; “+” = Increase.

“N” indicates a percentage change with absolute value <10%.

## 4 Discussion

In this study, different types of LSTV models were reconstructed based on data from a single healthy male volunteer. This approach was primarily adopted due to the high sensitivity of finite element analysis results to individual anatomical variations—differences in anatomical features and material properties across individuals (such as intervertebral discs, ligaments, and bony structures) can substantially affect the results. To eliminate interference from other confounding factors, we maintained consistent settings for intervertebral discs, ligaments, articular cartilage, and other bony structures, except for the key distinguishing features of different LSTV types (e.g., variations in the L5 transverse process and specific sacral structures). By applying physiological loads (a 400 N compressive force to simulate gravity and an 8.0 Nm torque to simulate flexion, extension, lateral bending, and rotation, respectively), we analyzed the biomechanical differences between the normal lumbosacral-pelvic structure and seven LSTV models classified by the Castellvi system. The results showed that in terms of overall displacement (Figure 5), Types ⅠA, ⅠB, ⅡA, and ⅡB exhibited no significant differences from the normal model under flexion and extension, while Type ⅠA only showed a slight difference under lateral bending. Notably, Types ⅠB, ⅡA, and ⅡB presented a mild reduction in displacement under pure compression, lateral bending, and rotation. This is consistent with their minor anatomical changes: Type Ⅰ (unilateral/bilateral elongation of the L5 transverse process) and Type Ⅱ (unilateral/bilateral synovial joint formation between the L5 transverse process and sacrum without a bony bridge) retain most of the normal integrity of the lumbosacral motion segment. In contrast, Types ⅢA, ⅢB, and Ⅳ differed from the normal model under all conditions, with a significant reduction in displacement. This is attributed to the bony continuity in Types Ⅲ and Ⅳ: unilateral/bilateral bony bridges exist between the L5 transverse process and sacrum, transforming the originally mobile L5-S1 segment into a rigid unit and restricting intervertebral rotation and translational movement.

In terms of the maximum Mises stress of the intervertebral disc, Types ⅢB and Ⅳ exhibited a significant reduction under all conditions. Among them, Type ⅢB showed the most obvious decrease (75.78%–96.11%), while Type Ⅳ decreased by 45.12%–90.47%. This rigid osseous fusion structure may transfer the load from the L5-S1 intervertebral disc to surrounding structures. For Type ⅢA, except under pure compression, the stress was reduced by 14.27%–86.39% under flexion, extension, lateral bending, and rotation, with an 86.39% reduction under left lateral bending. This is presumably due to the asymmetric load distribution caused by the unilateral bony bridge: the fused side bears more load (thereby reducing disc stress), while the non-fused side still retains part of the disc’s load-bearing function, which also explains the non-significant stress difference under pure compression and only a 14.27% stress reduction in the disc during right lateral bending. Types ⅠB and ⅡB showed consistent stress changes, with no significant differences from the normal model under flexion and rotation, but significant stress reductions under compression, extension, and lateral bending. Notably, Types ⅠA and ⅡA exhibited increased stress under flexion and rotation, which may be related to their asymmetric anatomical variations. Such asymmetry disrupts the normal load distribution between the disc and the posterior structures, leading to local stress concentration in the disc. Regarding the maximum Mises stress of the sacroiliac joints, Types ⅢB and Ⅳ showed little change in the bilateral sacroiliac joints under extension, but varying degrees of increase under other loading conditions, with the most significant increase under flexion. This may be because the rigid fusion of L5-S1 eliminates the shock-absorbing function of the intervertebral disc, forcing the sacroiliac joints to bear excessive axial loads and torsional loads; the sacroiliac joints themselves have limited mobility and cannot compensate for the lost motor function of the L5-S1 segment, ultimately leading to stress concentration on the surface of the joint cartilage. The sacroiliac joint stress of Type ⅢA presented an asymmetric characteristic of “increased on the left and decreased on the right”, which is directly related to the unilateral bony bridge structure of this type—the left fused side transfers more load to the ipsilateral sacroiliac joint, while the right non-fused side relies on the intervertebral disc to disperse the load, thereby reducing the sacroiliac joint stress. In contrast, the bilateral sacroiliac joint stress of Types ⅠB and ⅡB decreased under all conditions, which may be due to their slight structural changes (such as the formation of synovial joints between bilateral transverse processes and the sacrum without bony bridges)—these changes enhance lumbosacral stability without rigidifying the motion segment, achieving a balanced load distribution between the intervertebral disc and the sacroiliac joints.

The results of this study supplement and expand previous biomechanical studies on LSTV. Earlier finite element studies on LSTV (Zhu et al., 2025) mainly focused on Castellvi Type Ⅰ, finding that the maximum Mises stress of the L5-S1 intervertebral disc in Type Ⅰ LSTV did not increase significantly and even decreased slightly—this result is similar to that of Type ⅠB LSTV in the present study but slightly different from that of Type ⅠA. The main reason is that previous studies did not further subdivide Type Ⅰ into subtypes ⅠA and ⅠB, nor did they provide detailed biomechanical analysis of other LSTV subtypes. Through further research, this study revealed that different subtypes of Type Ⅰ and Type Ⅱ LSTV exhibit unique biomechanical effects: subtypes ⅠA and ⅡA (unilateral anatomical variation) cause increased disc stress under specific movement conditions due to structural asymmetry, while subtypes ⅠB and ⅡB (bilateral non-osseous fusion) reduce the stress of both the intervertebral disc and sacroiliac joint. These findings address the limitations of previous studies (Carapuço et al., 2005) and provide a potential biomechanical explanation for the controversial association between LSTV and discogenic low back pain observed in prior clinical research. Clinically, some studies have confirmed a correlation between LSTV and discogenic low back pain (Apaydin et al., 2019; Ravikanth and Majumdar, 2019; Gopalan and Yerramshetty, 2018), while others have failed to identify a significant relationship (Illeez et al., 2022; Luoma et al., 2004). This discrepancy may stem from the lack of stratification by Castellvi classification in previous investigations, which overlooked the biomechanical heterogeneity induced by anatomical differences among various LSTV subtypes. It is important to clarify that the mechanical findings of this study do not directly confirm a clinical causal relationship between LSTV subtypes and low back pain. Instead, they reveal the potential biomechanical basis by which different subtypes may indirectly influence the risk of intervertebral disc degeneration and pain through altering lumbosacral load distribution and stress transmission patterns. This provides a theoretical reference for subsequent targeted clinical stratified studies (e.g., analyses of associations between specific subtypes and pain incidence). Accordingly, this serves as the rationale for selecting L5-S1 intervertebral disc stress as a reference variable. Elevated disc stress may increase the risk of intervertebral disc herniation, providing a basis for formulating targeted preventive strategies (e.g., postural guidance) for high-risk LSTV subtypes. Additionally, overall model displacement and bilateral sacroiliac joint stress were included as reference variables. Overall displacement reflects lumbopelvic stability (Pel et al., 2008); an increase in this parameter indicates lumbopelvic instability, which may result in persistent strain on pelvic ligaments and subsequently induce low back pain and injury (Pool-Goudzwaard et al., 1998; Gale et al., 2018). Bilateral sacroiliac joint stress serves to assess bilateral sacroiliac joint function, and elevated bilateral sacroiliac joint stress may increase the risk of sacroiliac joint dysfunction/sacroiliitis (Zhang et al., 2022). By analyzing these biomechanical indicators, we aim to provide a reference for the prevention and treatment of high-risk LSTV subtypes.

In addition, a previous study (Zhu et al., 2025) also found that the maximum Mises stress of the upper adjacent disc (L4/5) was significantly increased in patients with Castellvi Type Ⅰ LSTV, suggesting that this type of anatomical variation may increase the risk of degeneration of the upper adjacent disc. Meanwhile, a cadaveric anatomical study (Aihara et al., 2005) observed that in cadaveric specimens with LSTV, the iliolumbar ligament in the upper segment was slimmer and structurally less stable than that in specimens without LSTV; the weakness of the iliolumbar ligament leads to reduced stability of the upper vertebral segment of the transitional vertebra, which may further induce subsequent disc degeneration—this provides a biomechanical and anatomical explanation for the clinical results reported by Apaydin et al. (Apaydin et al., 2019). Although the present study did not directly analyze the stress of the upper adjacent disc, combined with the characteristic of rigid fusion at the L5-S1 segment in Types Ⅲ and Ⅳ, it can be inferred that these subtypes may further aggravate the stress burden on the L4/5 disc through “upward load transmission”, a speculation supported by other studies (Cheng et al., 2022). That study confirmed that Castellvi Types Ⅱ, Ⅲ, and Ⅳ LSTV also increase the risk of degeneration of the upper adjacent disc, and the risk of L5-S1 disc degeneration in Types Ⅲ and Ⅳ is lower than that in the normal population—this is consistent with the result of significantly reduced L5-S1 disc stress in Types Ⅲ and Ⅳ in the present study.

Furthermore, the increased stress in the bilateral sacroiliac joints of Types ⅢB/Ⅳ (and the fused side of Type ⅢA) observed in this study provides a biomechanical basis for the clinical finding that “LSTV is associated with sacroiliitis” (Carvajal et al., 2020; Morbée et al., 2023; Kaya and Kerimoğlu, 2021). In addition, previous clinical studies did not conduct subtype analysis of LSTV, making it impossible to identify which subtype is more likely to induce sacroiliitis, while the results of the present study suggest that Types Ⅲ and Ⅳ may be high-risk subtypes for sacroiliitis. Furthermore, the elevated bilateral sacroiliac joint stress observed in Type ⅢB/Ⅳ LSTV (and on the fused side in Type ⅢA) in the present study appears to provide a biomechanical theoretical reference for the clinical finding that “LSTV is associated with sacroiliitis” (Carvajal et al., 2020; Morbée et al., 2023; Kaya and Kerimoğlu, 2021). Carvajal et al. (2020) found that LSTV increases the risk of sacroiliitis in patients with axial spondyloarthritis-related back pain; however, this study did not elaborate on the specific mechanisms. Instead, they proposed a speculative direction, suggesting that it may be related to altered lumbosacral biomechanical environments or impacts on load transmission in the bilateral sacroiliac joint. Notably, previous clinical studies did not conduct subtype analysis of LSTV, making it impossible to identify which subtype is more likely to induce sacroiliitis, while the results of the present study suggest that Types Ⅲ and Ⅳ may be high-risk subtypes for sacroiliitis.

Currently, numerous critical unresolved issues remain in LSTV research. One such issue is the lack of a clear consensus on the mechanism by which LSTV causes low back pain; beyond the aforementioned intervertebral disc degeneration and sacroiliitis, there is also the view that an oversized L5 transverse process compresses nerve roots. Kanematsu et al. (2020) found that 64% of LSTV patients had compression of the 5th lumbar nerve caused by a nearthrosis, primarily attributed to the tendency for osteophyte formation and the development of new bone and synovium-like tissue between the L5 transverse process and sacrum in LSTV patients (Porter et al., 2014), which leads to compression of the 5th lumbar spinal nerve root. Shibayama et al. (2011) reported a case where a patient underwent spinal canal decompression after being misdiagnosed with lumbar disc herniation-induced nerve root compression; however, the patient’s sciatica did not significantly improve postoperatively, and selective angiography later confirmed that the compression was caused by an oversized transverse process of the transitional vertebra. Another contentious issue involves other influencing factors in LSTV patients, such as age and gender. Carvajal et al. (2020) stratified 688 enrolled low back pain cases by age and gender and found no significant association between the occurrence of low back pain and age or gender in LSTV patients. In contrast, Bayram et al. (2025), among 2,516 enrolled patients, observed that young female LSTV patients were more prone to low back pain induced by degeneration of the upper adjacent disc. Regarding gender differences in the distribution of LSTV types, Mahato (2011), through a study of 320 cadaveric specimens, found that L5-S1 accessory joints (Castellvi Type Ⅱ) and S1 lumbarization were more common in females, while L5 sacralization (fusion between the lumbar vertebra and sacrum, Castellvi Types Ⅲ/Ⅳ) was predominantly observed in males; they also found that unilateral variations of LSTV were more common than bilateral ones. However, Byvaltsev et al. (2023), among 1,243 diagnosed LSTV patients, reported that 61.2% had bilateral variations and 38.8% had unilateral variations, which contradicts the findings of Mahato et al. These controversies indicate that current understanding of the influencing factors of LSTV remains insufficient, and further verification through large-sample, multi-center studies is required.

This study analyzed and compared the biomechanical differences among different Castellvi classifications of LSTV, providing a biomechanical reference for the classification-based diagnosis and intervention of LSTV-related low back pain. For LSTV patients with concurrent low back pain, it may be necessary to prioritize the identification of subtypes using CT (to detect bone bridges) and magnetic resonance imaging (MRI, to evaluate intervertebral disc integrity). Among these, patients with Type ⅠA/ⅡA LSTV whose pain is triggered by forward flexion or rotation require focused evaluation for discogenic pathologies (e.g., disc herniation, annulus fibrosus tear). Patients with Type ⅢB/Ⅳ presenting with hip or thigh pain (consistent with sacroiliac joint-referred pain) may need sacroiliac joint provocative tests (e.g., Faber test) or MRI to rule out sacroiliitis. Additionally, due to the high risk of chronic sacroiliac joint degeneration, regular follow-up with X-ray or MRI is recommended annually. In contrast, patients with Type ⅠB/ⅡB experience minimal biomechanical disturbance, and given their favorable prognosis and relatively low lesion risk, the frequency of imaging re-examinations can be reduced.

Despite the progress achieved in this study, several limitations remain to be addressed in future research. First, the analysis was based on an idealized three-dimensional finite element model constructed from the CT data of a single healthy Asian male, without accounting for the impact of individual differences (e.g., age, body weight, bone mineral density, and degree of intervertebral disc degeneration) on biomechanical characteristics. In subsequent studies, multi-subject models can be established, with the integration of patient-specific geometric structures. Second, this study only simulated the static response under a single physiological load, whereas the movement of the human lumbosacral region is multi-condition and dynamic. Although static loading is suitable for analyzing basic mechanical characteristics, it cannot fully replicate dynamic movements in real-world scenarios (such as walking and lifting objects), where stress on the lumbosacral region fluctuates continuously. Future research may employ dynamic load simulations (e.g., walking and bending to lift objects) to more authentically reflect the biomechanical behavior of lumbosacral transitional vertebrae (LSTV) during daily activities. Simulating dynamic scenarios may reveal stress concentration patterns that are inaccessible via static loading — for instance, the cumulative stress effect on the intervertebral discs above the LSTV. This finding may further validate the association between mechanical stress and the risk of related symptoms. Third, the model was simplified in certain aspects, such as the exclusion of surrounding muscles and the simplification of ligaments. Omitting muscle factors may underestimate the stabilizing effect of active muscle forces on the spine, potentially leading to minor deviations in the calculated stress values — though the overall trend of stress distribution remains consistent with clinical observations. Due to the significant nonlinear material properties of muscles, the nature of the muscle’s intrinsic reflex system, and cerebral control over muscles (Chang et al., 2022), simulating the properties and functions of muscles in current finite element analysis remains challenging. With the advancement of finite element technology in the future, more accurate simulation of the characteristics of muscle tissue and ligaments will be feasible. Fourth, this study focused on the L5-S1 segment and sacroiliac joints, while LSTV may exert effects on adjacent segments (e.g., L4-L5). Future research should extend the model to the L1-S2 segment to analyze stress changes in the upper lumbar intervertebral discs.

## 5 Conclusion

This study analyzed the biomechanical characteristics of seven Castellvi-classified LSTV types via finite element analysis, clarifying the load transmission patterns of different subtypes and their biomechanical differences in intervertebral discs and sacroiliac joints. Among these subtypes, Types ⅢB and Ⅳ significantly reduced intervertebral disc stress while increasing sacroiliac joint stress (including the left fused side in Type ⅢA), which elevates the risk of sacroiliac joint dysfunction (e.g., sacroiliac joint subluxation or sacroiliitis). Types ⅠA and ⅡA, due to increased local intervertebral disc stress and uneven stress distribution, may easily lead to discogenic low back pain. Types ⅠB and ⅡB caused minimal disturbances in overall displacement, intervertebral disc stress, and sacroiliac joint stress, leading to a relatively low risk of low back pain. These findings provide a biomechanical reference for the subtype-specific diagnosis and intervention of LSTV-related low back pain.

## Data Availability

The raw data supporting the conclusions of this article will be made available by the authors, without undue reservation.
